# Supplementary material for: Safety and contribution of elderly whole blood donors after raising the upper age limit: Hemovigilance data from a Chinese region from 2012 to 2023
Source: PLoS One. 2025 Sep 10;20(9):e0331838. doi: 10.1371/journal.pone.0331838 (PMC12422449; doi:10.1371/journal.pone.0331838)
Supplement: Attachment — Submitted filename: Response to Reviewers.docx [file pone.0331838.s003.docx]

**Evaluation of the safety and contribution of elderly whole blood donors after raising the upper age restriction: a study of donor hemovigilance data in a region of China from 2012 to 2023**

PONE-D-25-08734

Dear editor and reviewers:

We would like to thank the editor and all reviewers for your time and efforts in reviewing our manuscript entitled “Evaluation of the safety and contribution of elderly whole blood donors after raising the upper age restriction: a study of donor hemovigilance data in a region of China from 2012 to 2023”. Your comments and suggestions are valuable and helpful for revising and improving our paper, as well as important guidance for our studies in the future. Here, we have revised and re-submit the manuscript for your consideration. We have responded to each point brought up in your comments, and the changes are highlighted in the revised manuscript as well.

**Reviewer #1:**

This is an interesting study investigating the effect of allowing Chinese donors aged 55-60 to donate. As the authors state it is of great importance to conduct such studies to ensure donor health and monitor the effects of changes in donor eligibility criteria.
While I like the study, there are some things that I find are missing before it is ready for publication:

1. I lack a description of the study population in table form including average number donations, age, gender distribution ect.

**Response:**

Thank you for your attention to it. In the revised manuscript, we modified the content of Table 1 to feature information such as the age, gender and average blood donation volume of blood donors from 2012 to 2023. The contribution of elderly blood donors to the blood supply is visualized in Figure 1.

2. Are the older donors in this study new or experienced donors? And how does this differ from the younger group they are compared to? This should be accounted for in the analyses and in the interpretation of the results in the discussion.

**Response:**

Thank you for your nice suggestion. We added this part in the limitations of the discussion section of the revised manuscript. The content is as follows: In China, blood donors aged 56 to 60 are all experienced donors, because only those with blood donation experience can participate in blood donation after the age of 55. Therefore, such demographic characteristics will have a certain impact on the outcome, but it cannot be resolved within the current regulatory framework.

3. A clear description of the statistical methods is missing including how data was treated, models used and software.

**Response:**

Thank you for your advice. In the Materials and Methods section, we have added a description of the statistical methods: All data is exported from the blood information management system after approval. At the time the authors obtained the data, donor information had been anonymized. The authors obtained information during or after data collection that could not identify individual participants. R software (version 4.2.3) was used for all statistical analyses..GraphPad software (GraphPad Prism 9) was used to produce statistical graphs. The comparison of rates was conducted using the chi-square test, and a P value less than 0.05 was considered statistically significant.

4. For the VVR study it is not listed which covariates were included in the logistic(?) re-gression model. Number of donations or some measure of donation experience should be included to account for the healthy donor effect.

**Response:**

Thank you for your suggestion. Since this article mainly explores the impact of age on the safety of blood donors, we only compared the incidence of VVRs among blood donors of different age groups in the text to assess the safety of elderly blood donors, and did not conduct a regression analysis. As per your suggestion, we have supplemented the total number of blood donations and the specific data of VVRs in the results section.

5. The healthy donor effect and how this may impact results is not discussed or mentioned which I find strange.
   **Response:**

Thank you for your reminder. We added the content of the healthy blood donor effect in the discussion section of VVRs. Blood donors aged 56-60 are experienced donors, and the donors themselves have declared that they have not experienced any serious adverse reactions during previous blood donation procedures. This indicates that these donors have trust and confidence in the blood donation process, which also has a certain impact on the results.

6. Others
(1) Introduction
    Line 53-61: I lack conclusions on the findings from these manuscripts and why they have been mentioned. Also reg. ref. 4+5 please change to last name of the first author instead of first name (Joanna and Franke).

**Response:**

Thank you for your reminder. In this presentation, the mention of these studies is intended to illustrate that the health of elderly blood donors is receiving increasing attention. Of course, we have omitted the main conclusions of these studies. Following your suggestion, we have added the conclusion that elderly blood donors contribute more to blood supply and that blood safety is ensured. The following conclusions are drawn from these studies: On one hand, elderly blood donors have a stronger willingness to donate blood and their contribution to blood supply is increasing; on the other hand, no adverse effects on the health of elderly blood donors have been found due to blood donation behavior. The citation format of the research has been revised in the corresponding content in the article.

(2) Methods
Line 89-93: Should be in a separate section covering all methods sections.

**Response:**

We placed these contents in the section of statistical analysis methods.

Line 95-98: What is donation frequency limits in China? Please include this.

**Response:**

We have added the requirements of Chinese regulations on the frequency of blood donation here. The Blood Donation Law of China stipulates that the interval between two donations of whole blood should be no less than six months.

Line 113: Please include which assays are used, company etc..

**Response:**

We have added the description of statistical methods and included this part.

(3) Results
Table 1 should be cohort descriptives and the current table 1 would be easier to comprehend as a figure.

**Response:**

It has been modified as required.

Reviewer #2: Population aging has two impacts on the blood supply. On the one hand, fewer people are eligible to donate blood. On the other hand, the demand for clinical blood use has increased. In recent years, the feasibility of expanding elderly blood donors has been evaluated all over the world.

The problem is particularly prominent in China, a country that accounts for about 20% of the world's population, where the blood donation rate per thousand people was only 12.2‰ in 2024. Faced with the continued pressure on blood supply, the Chinese government relaxed the age limit for blood donors in 2012, expanding the age limit from 55 to 60 years in an effort to improve the blood availability.

However, blood is supplied to clinical patients, so its safety is of vital importance. This safety is reflected in two aspects: first, the safety of blood donors - as an elderly group, whether donating blood will bring them health risks; second, safety of blood products - whether the blood from the elderly can meet the quality requirements of transfusion medicine.

This paper is a retrospective analysis based on these two concerns. In general, the topic is meaningful, the logical structure of the writing is clear, and the conclusions are presented in an appropriate fashion and supported by the data. As an attempt of China's solution, it provides effective data support for solving China's blood supply problem, and also provides important reference for global countries.

However, the paper needs to be improved in the following aspects.

Major revision：

1. Written in standard English

Many expressions in this article do not meet the requirements of standard English. It is recommended to seek a native speaker for revision. The expressions that may cause ambiguity, unclear meaning, or sentences that are too long and need to be re-sentenced include but are not limited to:

Title, L26-30, L34-35, L54-61, L64-65, L69: ecology, L70: opposing, L74-75, L88-89, L91-92, L98: increase, L102: content, L186-190, L193: promote, L201: considers, L227-L234, L239: partly, L240, L244: lipid donation, L246: hypertriglyceridemic, L244-246, L251: diet, L252: TG, L257: suitability, L281&282: strengths.

**Response:**

Thank you for your suggestion. After we revised the manuscript, we invited a language editor from an English-speaking country to revise the language of the entire text and make detailed modifications to the content of the specific positions you proposed. Thank you again for your patient guidance, which has been of great help in improving the quality of our manuscripts. The title of the manuscript has been revised to “Safety and Contribution of Elderly Whole Blood Donors After Raising the Upper Age Limit: Hemovigilance Data from a Chinese Region from 2012 to 2023”.

2. Terminology for transfusion medicine

Many of the terms related to transfusion medicine in this article should be standardized, such as “donation” vs donor”, “donation” vs “donation times”. The mixed use of these expressions is particularly reflected in Table 1, L208, L212-218, etc.

Another example is the inconsistency between the "TTDI" in the main text and the title of Table 5. It is recommended to unify it as TTI (transfusion-transmitted infection).

**Response:**

Thank you for your reminder. According to your suggestion, we have standardized the terms of the entire text. The "donations" in this article is synonymous with the "donation times" you mentioned.

3. A statistical description should be added to the Materials and Methods section.

There are many statistical inference conclusions in the results section of the article, and the methodological information of these statistical analyses should be given in the materials and methods section.

In addition, “significantly” should be used with caution, and it needs to be supported by statistical inferences, such as in the "The deferral rate of elderly donors" section.

And, does the word “lower” in L167 mean statistically lower? Such expressions should be standardized.

**Response:**

Thank you for your advice. In the Materials and Methods section, we have added a description of the statistical methods: All data is exported from the blood information management system after approval. At the time the authors obtained the data, donor information had been anonymized. The authors obtained information during or after data collection that could not identify individual participants. R software (version 4.2.3) was used for all statistical analyses..GraphPad software (GraphPad Prism 9) was used to produce statistical graphs. The comparison of rates was conducted using the chi-square test, and a P value less than 0.05 was considered statistically significant.

Minor revision：

1. Regarding charts: It is recommended that at least Figure 2 be in color.

**Response:**

Thank you for your advice. We have changed Figure 2 to a color figure as per your suggestion.

2. Industry standard should be given a standard number, such as “GB 18469-2012”.

**Response:**

Thank you for your suggestion. We have added standard numbers to all the contents involving the names of national/industry standards in the article.

3. The age-related changes in triglyceride levels described in [23] do not appear to be sufficient to threaten the requirement for blood donation. It is necessary to carefully examine whether appropriate references have been cited. Other literatures should also be identified.

**Response:**

Thank you for your reminder. The relationship between plasma triglyceride levels and blood donation was indeed not mentioned in the references. The content of triglycerides can affect the appearance of plasma. Age changes can lead to changes in plasma triglyceride levels. Plasma TGs levels are higher in older adults versus younger adults. So we use the relationship between age and plasma triglyceride levels to explain the relationship between the age of blood donors and the appearance of plasma turbidity.

4. Others

L132-133: “percent” should be “%”, and L138: “II” should be “2”.

L273: “Le Chang et al” should be “Chang et al”, L275: “Ling Shiet al” should be “Shi et al”. L293: “stations” should be “banks”. L343, L370 should be the “National Health Commission of the People's Republic of China” or the “Former Ministry of Health of the People's Republic of China”.

**Response:**

Thank you for your suggestion. We revised the corresponding contents in the article.
