# Supplementary material for: Safety and contribution of elderly whole blood donors after raising the upper age limit: Hemovigilance data from a Chinese region from 2012 to 2023
Source: PLoS One. 2025 Sep 10;20(9):e0331838. doi: 10.1371/journal.pone.0331838 (PMC12422449; doi:10.1371/journal.pone.0331838)
Supplement: Attachment — Submitted filename: Response_to_Reviewers_auresp_2.docx [file pone.0331838.s004.docx]

**Evaluation of the safety and contribution of elderly whole blood donors after raising the upper age restriction: a study of donor hemovigilance data in a region of China from 2012 to 2023**

PONE-D-25-08734R1

Dear editor and reviewers:

We would like to thank the editor and all reviewers for your time and efforts in reviewing our manuscript entitled “Evaluation of the safety and contribution of elderly whole blood donors after raising the upper age restriction: a study of donor hemovigilance data in a region of China from 2012 to 2023”. Your comments and suggestions are valuable and helpful for revising and improving our paper, as well as important guidance for our studies in the future. Here, we have revised and re-submit the manuscript for your consideration. We have responded to each point brought up in your comments, and the changes are highlighted in the revised manuscript as well.

Reviewer #1:

1. Abstract: The conclusion section in the abstract would benefit from a clearer and more pre-cise interpretation e.g donor health = VVR risk.

**Response:**

Thank you for your suggestion. We have added an explanation of donor health in the methods section of the abstract and rewritten the conclusion content according to your suggestions to make the description more accurate.

Main text:
Methods:

2. Can you please clarify when and how ALT and turbidity are measures before dona-tion? Are the results available before the donation or performed on a pre-donation sample?

**Response:**

Thank you for your reminder. After the health consultation for blood donors was completed, we collected a sample to quantitatively and rapidly test the ALT and plasma turbidity of each donor. The doctor determined whether the blood donation would be delayed based on the test results. We have added these contents to the method.

3. For the blood safety methods section can you describe how testing is performed (equipment, is NAT(?) testing performed individually or in pools)? And in the corresponding results (line 101)section briefly mention how you define positive reactivity here? Is it solely NAT test posi-tive or also isolated antibody positive?

**Response:**

Thank you for your nice advice. The blood samples of donors were simultaneously tested by serological enzyme-linked immunosorbent assay (ELISA) and viral nucleic acid test (NAT). NAT testing performed individually by Tigris (Grifols). Blood with reactive ELISA or NAT test results was judged as unqualified.

4. Discussion: The first section of the discussion is very long and should either be drastically shortened or moved to the introduction. Please start the discussion by summarizing your main findings in a short and precise manor. Likewise, the conclusion section is lacking the main findings and why the authors conclude it’s safe to include elderly donors.

**Response:**

Thank you for your suggestion. We rewrote the first part of the discussion. Some content has been streamlined and a brief description of the main results has been added. The conclusion section has also been rewritten.

5. Deferral results in the discussion: It’s interesting and counterintuitive that older donors have higher plasma turbidity deferrals but fewer ALT deferrals when ALT can be moderately in-creased by dyslipidemia and obesity. Do donor selection criteria use the same cutoff for all donors, or an age adjusted one? I miss a discussion of this and if current selection criteria are suitable for older donors.

**Response:**

Thank you for your detailed review and interesting questions. Yes, all blood donors are judged by the same criteria for plasma turbidity. Because the standard for blood distribution is the same and will not change due to the different ages of the blood donors.

Specific comments:

6. Figures 3+5: I would suggest adding * or similar when significant difference between the groups

**Response:**

Thank you for your suggestion. We made modifications to Figures 3 and 5, adding annotations with statistical differences.

7. Line 207: Please refer to the latest blood guide (22nd edition) and the age criteria there, this is outdated and should not be used.

Thank you for pointing out the error in this version of the literature. To streamline the first part of the discussion, we deleted this reference.

8. Line 290: Why does it impact the results? I would suggest referring to a paper on the healthy donor effect or simply state it here.

**Response:**

Thank you for pointing out the shortcomings here. We made supplementary explanations in this discussion. Experienced blood donors are familiar with the blood donation process, which can reduce their fear of donating blood and thereby decrease the occurrence of VVRs.

Reviewer #2: 
I Written in standard English
1. L66-67: “Notably, the safety of blood donors, as well as the collected blood, should be closely monitored”.I can’t understand the meaning of this sentence, especially “collected blood”.
**Response:**

Thank you for your suggestion. We rewrote this sentence. The safety of elderly blood donors and the blood they donate needs to be given attention.

2. L98: “increase” limit?

**Response:**
Thank you for your reminder. We rewrote this sentence in the revised manuscript. These findings show the contribution of elderly blood donors to blood supply following the raising of the upper age restriction of blood donors.

3. L207-210: Why use the past tense?
By the way, it is currently in its 22nd edition. And the file name is “Guide to the preparation, use and quality assurance of blood components” instead of “Guidelines on the Preparation, Use and Quality Assurance of Blood Components”.

**Response:**

Thank you for pointing out the error in this version of the literature. To streamline the first part of the discussion, we deleted this reference.

4. L247-248: At least, “the rate of deferred blood donation and the causes of deferred blood donation” can be: “the rate and causes of deferred blood donation”.
In addition, there are rarely expressions like “at various ages”.

**Response:**

Thank you for your suggestion. We made corrections in the revised manuscript.

5. L271: Can “poorer diet” cause “high lipid levels”?

**Response:**

Thank you for your reminder. Here it refers to poor dietary habits. We made corrections in the revised manuscript.

6. L273-274: What’s TG?
Acronyms and abbreviations must be spelled out completely on initial appearance in text.
**Response:**

Thank you for your suggestion. Here, TG refers to triglycerides. We marked the position where it first appeared.

7. L303: It is recommended to: Highlights and limitations of the study.
**Response:**

Thank you for your recommendation. We have adopted your recommended content in the revised manuscript.

II Others
8. L166-167: Figure 3 does not illustrate “the deferral rate was lower for older male donors than for younger donors”, or quantitative data such as 1.50%, 1.76%, etc.
In particular, the “Hb” and “Plasma turbidity” of the older donors were significantly higher than those of the younger donors.
The same goes for female (the “Hb” and “Other deferrals” of the older donors were significantly higher than those of the younger donors).
**Response:**

Thank you for your suggestion. We made modifications to Figures 3 and 5, adding annotations with statistical differences.

9. L273-274: Reference 23 discusses how alterations in triglyceride metabolism contribute to metabolic diseases, but does not mention the impact on plasma appearance.
**Response:**

Thank you for your suggestion. We have cited this literature here to illustrate the influence of age on triglyceride metabolism. The concentration of plasma triglycerides can affect the turbidity of plasma.

10. L403: Should be: “National Health Commission of the People’s Republic of China” or “Former Ministry of Health of the People’s Republic of China”.
**Response:**

Thank you for your suggestion. We made corrections in the revised manuscript.

11. There is a lack of citation of reference [17].
All the references should be reviewed.
**Response:**

Thank you for your careful review. We made corrections in the revised manuscript and reviewed the references throughout the text.

12. Since P has been specified in the materials and methods section, it should be uniform throughout the main text.

**Response:**

We reviewed the full-text statistical description.

13. “previous blood donor” and “experienced donor” should be unified as “repeat donor”.

**Response:**

Thank you for your suggestion. We adopted the expression you suggested in the revised manuscript.
