# Supplementary material for: Safety and contribution of elderly whole blood donors after raising the upper age limit: Hemovigilance data from a Chinese region from 2012 to 2023
Source: PLoS One. 2025 Sep 10;20(9):e0331838. doi: 10.1371/journal.pone.0331838 (PMC12422449; doi:10.1371/journal.pone.0331838)
Supplement: Attachment — Submitted filename: Response_to_Reviewers_auresp_3.docx [file pone.0331838.s005.docx]

**Response to Reviewers**

Evaluation of the safety and contribution of elderly whole blood donors after raising the upper age restriction: a study of donor hemovigilance data in a region of China from 2012 to 2023

PONE-D-25-08734R3

Dear editor:

Thanks very much for your kind work of our manuscript entitled “Safety and Contribution of Elderly Whole Blood Donors After Raising the Upper Age Limit: Hemovigilance Data from a Chinese Region from 2012 to 2023”. On behalf of my co-authors, we would like to express our great appreciation to editor and reviewers. At your suggestion, we have revised this manuscript and the new revised version has been completed. After the revision, the quality of the manuscript has been significantly improved. We have responded to each point brought up in your comments, and the changes are highlighted in the revised manuscript as well.

In addition, we previously expressed to you via email my request to add another author and received your consent. We would like to express our gratitude once again for your help. In this revised manuscript, we have added Xiaobing Zhu as a co-corresponding author.

1. **Abstract**

Thid is much improved - please change line 31 to say the overall total proportions of elderly blood donations increased 10-fold. 'All blood donations' is not the correct term as you only examining donors who perform whole blood donation. All blood donations imply other types of donations such as plasma and platelets as well as whole blood.
**Response：**

Thank you for your suggestion. There was indeed an imprecise expression here. We have made the revision based on your advice. In the revised manuscript, 'All blood donations' has been changed to "whole blood".

**2. Introduction**
line 67 remove extra full stop
please consider moving lines 71-74 before line 67 'current blood transfusion.....'
**Response：**

Thank you for your careful review of the manuscript. We have removed this unnecessary punctuation mark in the revised manuscript and reordered the content according to your requirements.

**3. Methods**
line 95 - please put here the allowable donation frequency for your centre?
is the last allowable age when each donor turns 60? If so, this should be stated.
line 106 - which exact test is used to perform plasma turbidity and ALT or place any references for these tests. what was the readout values you used and please state that the same values were used for every donor regardless of age.
line 121 if NAT is unqualified how is it later confirmed to be positive. Are results from additional testing returned to the donor centre? If not, then possibly consider saying in your results about the TTID that they are not positive but unconfirmed reactives?
**Response：**

Thank you for your reminder. We have made the following revisions in the revised manuscript we submitted:

line 95 – Add “the interval between two donations should be no less than 6 months”.

Not all blood donors are allowed to extend to the age of 60. Only repeat donors can be extended to 60, while first-time donors still have the limit of 55. This information was explained in the final paragraph of our introduction.

line 106 – We utilized QL1000C Automatic Blood Donation Screening Analyzer (Hope Medical) to measure plasma turbidity and ALT levels. The assessment of plasma turbidity was conducted using turbidity analysis, which categorizes the degree of turbidity into three grades—Grade I (mild), Grade II (moderate), and Grade III (severe). Blood donation is permitted for donors with plasma turbidity classified as Grade I or Grade II. ALT levels were determined using the rate method, and donors are eligible to donate blood if their ALT level is ≤ 50 U/L.

line 121- Regarding the TTID results, we did not conduct a confirmatory test to determine if they were positive; instead, they were unconfirmed reactive results.

4. **Results
   (1)** You provide limited p value data to show significance...please put these in next to your % value for each comparison whilst in the figure it should be in the main text and also put as a value if available rather than >0.05.

**Response：**

Thank you for your suggestion. We reprocessed the full-text statistical expression and presented all P-values.

(2) you never reference supplementary tables anywhere in the results. please put a reference to them where they are supposed to be viewed for readers.

**Response：**

Thank you for your reminder. We analyzed the results of supplementary table 1 in lines 160-164 and refer to supplementary table 1 here.

（3） line 140 - are there any significant differences in the total number of male and female donors analysed by age group? If so, these should be reported.

**Response：**

Thank you for your suggestions. On lines 148 to 150 of the revised manuscript. The number of blood donors aged 56-60 accounted for 0.83% of the total number of male and 0.73% of the total number of female blood donors respectively, and the difference was statistically significant (p <.000001).

（4） line 139-141 repeat of line 167-169...please check the manuscript carefully for repeated information

**Response：**

Thank you for your careful review. However, we have compared these two parts and found no duplicate content.

（5） Please consider removing figure 1, it does not seem helpful to your message. The data is clear from the presentation in table 1.

**Response：**

Thank you for your suggestion. We compared the content of Table 1 with that of Figure 1. Table 1 mainly shows the number of blood donors and the frequency of blood donation. Figure 1 mainly shows the trend changes in blood contributions made by blood donors aged 56 to 60 during the period from 2012 to 2023. They express different contents. After careful consideration, we suggest retaining Figure 1.

（6） in your supplementary table 1 you indicate the volume of each donation provided which is useful. Are there any differences of value that should be raised in the main text.

Please also reference supplementary table 1 in your manuscript.
Please check the file for supplementary table 1 to make sure only one sheet is given

**Response：**

Thank you for your suggestion. We have added the results of the data analysis in supplementary table 1 to the article (Lines 160-164). Among male blood donors, the proportion of those aged 56-60 who donated 400ml of whole blood was 83.72%, significantly higher than 70.10% for those aged 18-55, p < 0.000001. Similarly, among female blood donors, the proportion of those aged 56-60 who donated 400ml was 76.99%, significantly higher than 47.86% for those aged 18-55, p < 0.000001 (supplementary table 1).

(7) Does volume donated influence the VVR rates? I suspect there is some influence.

**Response：**

Thank you for your question. Yes, donating whole blood volume can affect the incidence of VVR. Our data analysis shows that blood donors aged 56 to 60 (both male and female) have a higher proportion of donating 400ml of whole blood, but the incidence of VVR is significantly lower than that of blood donors aged 18 to 55. We added the elaboration of this content in the discussion. (lines 296-299)

(8) line 182-285 is unclear what do you mean by small amount of vvr data?

**Response：**

Thank you for your careful review. There is indeed a problem of unclear expression here. We have deleted the relevant content.

(9) line 190 - can you say positive if you say they are unqualified in the methods? might be advisable to change to reactivity rate was higher?

**Response：**

Thank you for your suggestion. We have changed "positive" in the text to "reactivity".

(10) line 192-193 - what is the p value? doesn't seem significant? Therefore, it may not be higher?

**Response：**

We have added the description of the p-value in the text.

(11) line 197 says HIV screening reactivity if higher in younger donors but the figure shows opposite. please check
**Response：**

Thank you for your careful review and for pointing out our mistakes. There are indeed typos in the expression here. We have made corrections in the revised manuscript.

**5. Discussion**
it is long can you make some more concise statements and perhaps refrain from repeating the results once again. you should put your findings into context without repeating the % values found. for example you can clearly state that older females had higher reactivity to HIV. We theorise that this occurs due to....
line 206 - you should say 'adjusting donor age to 60 on blood donation health'
line 215 - you need to state that this was in older female donors
line 217-219 repeat of 210-212.
line 221-225 - this is due to allowable donation frequency though and the Australian rate was probably including plasma/platelet donation which increases the frequency
how have you told potential donors in age range 56-60 that they are eligible to donate? numbers may be small as they don't know they can donate?

line 239 - please specify young male donors in that line

**Response：**

Thank you for the good questions you raised. We revised each suggestion one by one. Indeed, we have not specifically informed every blood donor who is 55 years old or above that they can continue to donate blood until they are 60. It was only when these blood donors came for consultation that they learned they could continue donating blood. We added these contents in the discussion. (lines 243-246)

**6. supplementary files**
please check each file and only provide the sheet that has the information you want to use
supp table 2 - which bit of data are you trying to provide
supp table 3 - please move sheet 2 and 3. please don't use abbreviations. you should put the data together as a proper table not separate lines in the excel
supp table 4 - what value does this have as you already have it as a figure 4
supp table 5 - why have you put ALT again? please also make into a proper table even if presented in excel

**Response：**

Thank you for your suggestion. Our original understanding of the supplementary table was to provide the original data for drawing. Now we know that it only provides the content not shown in the text. Therefore, we have reorganized the supplementary table. Only supplementary table 1 was retained because the contents of the other appendices have already been reflected in the pictures.
